# Fractional ethanol precipitation modulates the structure and function of *Coprinus comatus* mycelial polysaccharides: alleviation of DSS-induced injury in Caco-2 cells via regulation of mitochondrial function

**DOI:** 10.3389/fnut.2026.1743246

**Published:** 2026-01-22

**Authors:** Ruijie Chen, Tao Gu, Zhun Xiang, Shaofeng Wei

**Affiliations:** 1 School of Public Health, The Key Laboratory of Environmental Pollution Monitoring and Disease Control, Ministry of Education, Guizhou Medical University, Guiyang, Guizhou, China; 2 Guizhou Academy of Sciences, Guizhou Provincial Institute of Biology, Guizhou Key Laboratory of Agricultural Microbiology, Guiyang, China

**Keywords:** CaCo-2, *Coprinus comatus*, edible fungal mycelium, fractional ethanol precipitation, mitochondria

## Abstract

**Introduction:**

This study aimed to systematically fractionate *Coprinus comatus* mycelial polysaccharides using ethanol precipitation, screen the optimal fraction, and investigate its potential to alleviate inflammation in a dextran sulfate sodium (DSS)-induced Caco-2 cell model.

**Methods:**

Polysaccharides (CCPJ-40, -60, -80) were obtained with 40%, 60%, and 80% ethanol. Their chemical properties (yield, monosaccharide composition, molecular weight) and in vitro bioactivities were assessed. The optimal fraction was investigated in a dextran sulfate sodium (DSS)-induced Caco-2 cell model.

**Results:**

The results revealed that the ethanol concentration significantly affected the polysaccharide yield, chemical composition, and bioactivities. Notably, CCPJ-80 exhibited the highest yield and total sugar content. Monosaccharide composition analysis indicated that both CCPJ-40 and CCPJ-80 were composed of L-fucose, L-arabinose, galactose, glucose, xylose, mannose, ribose, and D-glucuronic acid, whereas CCPJ-60 additionally contained rhamnose. Regarding molecular weight distribution, CCPJ-40 had a high molecular weight and was relatively polydisperse, while CCPJ-60 and CCPJ-80 had lower molecular weights with good homogeneity. In the *in vitro* activity assessment, CCPJ-80 demonstrated the most potent antioxidant and prebiotic activities. Further cell experiments confirmed that CCPJ-80 effectively alleviated the DSS-induced inflammatory state in Caco-2 cells. The underlying mechanisms included significantly reducing the levels of lactate dehydrogenase, tumor necrosis factor-α, interleukin-6, reactive oxygen species, and mitochondrial ROS, while simultaneously enhancing superoxide dismutase activity and restoring the mitochondrial membrane potential.

**Discussion:**

This study elucidates the regulatory effect of ethanol precipitation concentration on the physicochemical properties and functional activities of CC mycelial polysaccharides, and reveals the potential of CCPJ-80 to ameliorate ulcerative colitis by modulating oxidative stress and mitochondrial function pathways, providing a scientific basis for developing CCPJ as a functional food or therapeutic agent.

## 1 Introduction

Ulcerative colitis (UC) is a chronic, non-specific inflammatory bowel disease. Its pathological features typically originate in the rectum and can spread continuously to the proximal colon (1). The disease commonly presents with core symptoms such as bloody diarrhea, passage of mucopurulent bloody stools, and abdominal pain (2). Characterized by a long disease course, high recurrence rate, difficulty in treatment, and substantial impairment of quality of life, UC is often termed “green cancer” due to its persistent and challenging nature (3). It has emerged as a significant global public health challenge (4). By 2023, the global prevalence was estimated to be as high as approximately 5 million cases, and the incidence continues to show a sustained increasing trend (5). Current clinical management of UC patients primarily involves therapeutic agents such as sulfasalazine, mesalazine, glucocorticoids, and biological agents. However, patients often encounter challenges including inadequate response, side effects, and high costs (6).

Caco-2 cells spontaneously differentiate during *in vitro* culture, exhibiting characteristics of both small intestine and colon, which makes them a commonly used in vitro model for intestinal barrier research (7). Lu et al. (8) found that treating Caco-2 cells with *Porphyromonas gingivalis* outer membrane vesicles induced mitochondrial damage. Mitochondrial damage is recognized as playing a crucial role in the pathogenesis and progression of UC (9). Significant suppression of genes related to mitochondrial metabolism has been observed in the colorectal tissues of UC patients (10). Therefore, mitigating mitochondrial damage to protect the intestinal barrier, achieved by alleviating oxidative stress and inflammation (11), has emerged as a potential strategy for UC prevention and treatment (12). In recent years, natural bioactive compounds have shown promise for UC intervention (13). Among them, natural polysaccharides, which can ameliorate UC through multiple pathways such as enhancing the intestinal barrier, modulating oxidative stress, and suppressing inflammation (14), have become a prominent research focus.

*Coprinus comatus* (CC), commonly known as the shaggy ink cap, is a macrofungus valued for both its culinary and medicinal properties. It is recognized by the Food and Agriculture Organization and the World Health Organization as one of the 16 rare edible mushrooms possessing “natural, nutritional, and health-promoting” functions (15). CC is rich in various bioactive compounds, including functional substances such as polysaccharides, polyphenols, fatty acids, tocopherols, and organic acids (16). Among these, polysaccharides are considered the most critical bioactive components (17). Studies have demonstrated that *Coprinus comatus* polysaccharides (CCP) exhibit a range of physiological activities, including hypoglycemic (18), immunomodulatory (19), immunomodulatory (20), hypolipidemic (21), and antitumor effects (22). The mycelium serves as the vegetative organ of the fungus; without robust mycelial growth, high-quality fruiting bodies cannot be formed (23). Utilizing submerged fermentation to cultivate *C. comatus* mycelium for the extraction of *Coprinus comatus* mycelial polysaccharides (CCPJ) offers significant advantages: the production cycle is shortened, the process is simpler, raw materials are readily available, production costs are substantially reduced, and the total extraction yield of polysaccharides from the mycelium is higher compared to that from the fruiting bodies (24).

Fractional ethanol precipitation is an effective strategy for the separation and purification of polysaccharides. Compared to traditional extraction methods, it offers significant advantages including high separation efficiency, low cost, and ease of scale-up for industrial production (25). Leveraging these advantages, this study employed the method to fractionate CCPJ at different ethanol concentrations and systematically compared the differences in their physicochemical properties and bioactivities. Based on the structural analysis, the CCPJ fraction with the most favorable physicochemical properties was selected for further investigation into its potential ameliorative effects using a dextran sulfate sodium (DSS)-induced inflammatory model in Caco-2 cells. This study aims to elucidate whether CCPJ alleviates intestinal inflammation by improving mitochondrial function. The findings will provide a theoretical basis for the functional application of CCPJ in UC intervention and lay a foundation for the development and utilization of natural polysaccharides in the field of intestinal health.

## 2 Materials and methods

### 2.1 Materials

*Coprinus comatus* strain (Xuzhou, Jiangsu, China); Caco-2 cells (Zibin Bio, Wuhan, China); Potato Dextrose Agar (PDA) (Hope Bio, Qingdao, China); Glucose (Zhiyuan, Tianjin, China); 1,1-Diphenyl-2-picrylhydrazyl (DPPH) (Macklin, Shanghai, China); L-Ascorbic acid (Vc) (Macklin); Trifluoroacetic acid (TFA) (ANPEL, Shanghai, China); Methanol (ANPEL); Sodium hydroxide (NaOH) (Sigma-Aldrich); Sodium acetate (Sigma-Aldrich); 2, 2′-Azinobis (3-ethylbenzothiazoline-6-sulfonic acid) diammonium salt (ABTS) (Macklin); Absolute ethanol (Fuyu Reagent, China); Hydrogen peroxide (H₂O₂); Ferrous sulfate heptahydrate (FeSO₄·7H₂O) (Shanghai Hushi, China); Dulbecco’s Modified Eagle Medium (DMEM) (Gibco); Fetal Bovine Serum (FBS) (Gibco); 1% Penicillin–Streptomycin solution; CCK-8 assay kit (Beyotime, China); Reactive Oxygen Species (ROS) assay kit (Beyotime); Superoxide Dismutase (SOD) assay kit (Beyotime); Mitochondrial ROS (mtROS) assay kit (Solarbio, China); JC-1 Mitochondrial Membrane Potential Assay Kit (Solarbio); Interleukin-6 (IL-6) ELISA Kit (Solarbio); Tumor Necrosis Factor-alpha (TNF-*α*) ELISA Kit (Solarbio).

### 2.2 Preparation of CC mother culture and production of liquid inoculum

Under aseptic conditions, a 0.5 cm^2^ mycelial block of CC was inoculated onto a PDA slant agar medium and cultivated at 25 °C for 1 week. Under a laminar flow hood, a 0.5 cm^2^ mycelial block was aseptically excised from the fully colonized medium and inoculated into a conical flask containing 400 mL of PDA liquid medium. After static cultivation overnight at room temperature, the culture was transferred to a constant-temperature incubator shaker and incubated at 25 °C with agitation at 160 rpm for approximately 8 days. Mycelial pellets exhibiting robust growth and uniform size were selected and transferred to fresh PDA liquid medium for expanded cultivation.

Upon completion of the cultivation, the fermentation broth was separated and removed by vacuum filtration to collect the CC mycelium. The mycelium was thoroughly washed three times with distilled water and dried by vacuum filtration until no free water remained, yielding purified mycelial biomass. Finally, the mycelium was dried in an oven at 45 °C until a constant weight was achieved, and subsequently weighed.

### 2.3 Extraction and preparation of crude polysaccharides and calculation of extraction yield

The dried mycelium was removed, pulverized, and passed through a 40-mesh sieve for subsequent use. A 30 g sample of the mycelial powder was weighed, and distilled water was added at a solid-to-liquid ratio of 1:30 (g/mL). The mixture was treated in a water bath at 90 °C for 3 h. After extraction, the mixture was filtered and centrifuged at 5000 r/min for 20 min to collect the supernatant. The residue was re-extracted once under the same conditions, and the two resulting supernatants were combined.

The combined supernatant was concentrated by rotary evaporation to one-fifth of its original volume. Absolute ethanol was added to the concentrate to achieve an ethanol concentration of 40% (v/v). The solution was allowed to stand at 4 °C for 24 h, followed by filtration and centrifugation, to obtain CCPJ-40. Subsequently, absolute ethanol was added to the resulting supernatant to increase the ethanol concentration to 60% (v/v). After standing at 4 °C for 24 h, followed by filtration and centrifugation, CCPJ-60 was obtained. This process was repeated by adding absolute ethanol to the supernatant to reach a final ethanol concentration of 80% (v/v). Following standing at 4 °C for 24 h, filtration, and centrifugation, CCPJ-80 was obtained. The resulting precipitates were freeze-dried to yield the graded ethanol-precipitated crude CCPJ polysaccharides.

The total sugar content (n) in CCPJ was determined using the method described for total sugar content assay (26), and the polysaccharide extraction yield (W) was then calculated using the following formula:


W=(M∗n/Mt)∗100%

W is the crude polysaccharide extraction yield; M is the mass of the crude polysaccharide; n is the polysaccharide content in CCPJ; Mt. is the mass of the CCPJ powder.

### 2.4 Polysaccharide purification

Proteins were removed from the polysaccharide extract using the Sevag reagent (chloroform: n-butanol = 4:1 v/v) (27). The crude CCPJ powder was dissolved in an appropriate volume of distilled water and mixed with the Sevag reagent at a ratio of 4:1 (aqueous phase: Sevag reagent). The mixture was vigorously stirred for 30 min using a magnetic stirrer, followed by centrifugation at 5000 r/min for 3 min. The lower organic phase and the intermediate white insoluble layer (denatured proteins) were discarded. The Sevag reagent was added to the remaining aqueous phase at the same ratio, and this process was repeated until no white protein precipitate was observed at the interface. The deproteinized polysaccharide aqueous solution was then heated and concentrated to remove residual Sevag reagent.

The protein-free polysaccharide solution was dialyzed against distilled water for 72 h using dialysis tubing with a molecular weight cut-off (MWCO) of 8,000–14,000 Da. The water was changed every 6 h to remove small molecules such as monosaccharides. After dialysis, the polysaccharide solution was concentrated to a viscous consistency and subsequently freeze-dried under vacuum at −55 °C to obtain the purified CCPJ.

### 2.5 Structural analysis of CCPJ

#### 2.5.1 Monosaccharide composition

The analysis was performed with reference to the method described by Zhu et al. (28) The chromatographic conditions were as follows: A Dionex™ CarboPac™ PA20 analytical column (150 × 3.0 mm, 10 μm) was used. The injection volume was 5 μL. The mobile phases consisted of phase A (H₂O), phase B (0.1 M NaOH), and phase C (0.1 M NaOH, 0.2 M NaOAc). The flow rate was set at 0.5 mL/min, and the column temperature was maintained at 30 °C. The elution gradient program was: 0 min (95:5:0, A/B/C, v/v/v), 26 min (85:5:10, A/B/C, v/v/v), 42 min (85:5:10, A/B/C, v/v/v), 42.1 min (60:0:40, A/B/C, v/v/v), 52 min (60:40:0, A/B/C, v/v/v), 52.1 min (95:5:0, A/B/C, v/v/v), and 60 min (95:5:0, A/B/C, v/v/v).

#### 2.5.2 Absolute molecular weight

The absolute molecular weight was determined according to the method described by CHEN et al. (29) The sample was dissolved in a 0.1 M NaNO₃ aqueous solution (containing 0.02% NaN₃, w/w) to a final concentration of 1 mg/mL, filtered through a 0.45-μm membrane filter, and then injected for analysis.

The chromatographic conditions were as follows: A series of gel permeation chromatography (GPC) columns, Ohpak SB-805 HQ (300 × 8 mm) and Ohpak SB-803 HQ (300 × 8 mm), were used. The column temperature was maintained at 45 °C, the injection volume was 100 μL, and the mobile phase was 0.1 M NaNO₃ containing 0.02% NaN₃ (w/w). The flow rate was set at 0.6 mL/min with an isocratic elution for 75 min.

#### 2.5.3 Fourier transform infrared spectroscopy

A small amount of the polysaccharide sample was thoroughly mixed with 200 mg of potassium bromide (KBr) and pressed into a 1-mm-thick pellet for analysis. The analysis was performed using a Nicolet iZ-10 Fourier Transform Infrared Spectrometer. The instrumental parameters were set as follows: resolution, 4.00 cm^−1^; scan range, 4,000–450 cm^−1^; number of scans, 32; gain, 8.0; mirror velocity, 0.4747 cm/s; aperture, 80.00. The instrument was equipped with a DTGS KBr detector, a KBr beamsplitter, and an infrared source.

### 2.6 Analysis of antioxidant activity

#### 2.6.1 DPPH radical scavenging assay

Two milliliters of polysaccharide solutions at different concentrations (2, 4, 8, 12, and 20 mg/mL) were separately mixed with 2 mL of a DPPH solution (0.5 mmol/L). The mixtures were vortexed and allowed to react in the dark at room temperature for 30 min. The absorbance at 515 nm was then measured. Distilled water was used instead of the sample solution as the blank control, and ascorbic acid (Vc) was used as the positive control. The DPPH radical scavenging activity was calculated using the following formula:


$$\text{DPPH scavenging rate}\;(\%)=[1-(A-A_{0})/A_{1}]\times 100\%.$$

A is the absorbance of the mixture containing the polysaccharide sample (or positive control) and the DPPH ethanol solution. A₀ is the absorbance of the mixture containing the polysaccharide sample and ethanol (correcting for the sample’s intrinsic color). A_1_ is the absorbance of the mixture containing distilled water and the DPPH ethanol solution (representing the initial DPPH concentration).

#### 2.6.2 ABTS^+^• radical scavenging assay

A 1.0 mL aliquot of 7.4 mmol/L ABTS stock solution was mixed with 1.0 mL of 2.6 mmol/L K₂S₂O₈ stock solution. The mixture was allowed to stand in the dark at room temperature for 12 h to generate the ABTS^+^• radical cation. The resulting solution was then diluted with phosphate buffer (pH 7.4) to obtain the ABTS^+^• working solution. Subsequently, 0.4 mL of polysaccharide solutions at various concentrations (2, 4, 8, 12, and 20 mg/mL) were separately mixed with 4.0 mL of the ABTS^+^• working solution. After reacting in the dark at room temperature for 6 min, the absorbance was measured at 734 nm. The ABTS^+^• scavenging activity was calculated using the following formula:


$$\mathrm{ABT}S^{+}•\text{scavenging rate}\;(\%)=[(A_{0}-A)/A_{0}]\times 100\%$$

A₀ is the absorbance of the blank control (the ABTS^+^• working solution mixed with buffer or solvent). A is the absorbance of the mixture containing the polysaccharide sample or positive control and the ABTS^+^• working solution.

#### 2.6.3 Hydroxyl radical (OH) scavenging assay

Sequentially, 0.5 mL of polysaccharide solutions at various concentrations (1, 2.5, 5, 7.5, and 10 mg/mL) was mixed with 0.5 mL of FeSO₄ solution (6 mmol/L) and 0.5 mL of H₂O₂ solution (6 mmol/L). The mixture was vortexed and allowed to react for 10 min. Subsequently, 1.5 mL of salicylic acid solution (6 mmol/L) was added, and the reaction mixture was immediately transferred to a constant-temperature water bath at 37 °C for precisely 30 min. After the reaction, the absorbance was measured at 510 nm. Following the same procedure, the absorbances labeled A₁ for VC (positive control), A₂ for absolute ethanol (sample background control), and A₃ for distilled water (blank control) were measured. The hydroxyl radical scavenging rate was calculated using the following formula:

Hydroxyl radical (·OH) scavenging rate (%) = [1−(A₁−A₂)/A₃] × 100%

### 2.7 Growth promotion of *Lactobacillus plantarum*

*Lactobacillus plantarum* was activated in MRS broth medium with a 1% inoculation volume. After 12 h of cultivation, the bacterial suspension was diluted to a concentration of 10^6^ CFU/mL for subsequent use. The experiment was performed according to the method described by Tian et al. (30) with minor modifications. Briefly, bacterial suspensions of each treatment group were inoculated into a sterile 96-well plate. The groups included: a negative control (sugar-free MRS medium), a positive control (sugar-free MRS medium supplemented with inulin), and three experimental groups (sugar-free MRS medium containing CCPJ-40, CCPJ-60, or CCPJ-80, respectively). Both inulin and the polysaccharides in the experimental groups were added to the sugar-free MRS medium at a concentration of 20 mg/mL (as determined in preliminary tests). In each well, 50 μL of bacterial suspension (10^6^ CFU/mL) was mixed with 150 μL of the corresponding medium to obtain a final volume of 200 μL and an initial bacterial concentration of approximately 5 × 10^5^ CFU/mL. Each treatment group was set up in three replicate wells. The OD_600_ value was measured at 2-h intervals, and the growth curve was plotted accordingly.

### 2.8 Caco-2 cell culture and cytotoxicity assay

Caco-2 cells were cultured in DMEM medium supplemented with 20% fetal bovine serum (FBS) and 1% penicillin–streptomycin (P/S). The cells were seeded into 96-well plates at a density of 2 × 10^5^ cells/mL (100 μL per well) and cultured for 24 h. Subsequently, the cells were treated with CCPJ at concentrations of 0, 50, 100, 200, 400, 800, 1,600, 3,200, and 6,400 μg/mL, or with DSS at concentrations of 0, 0.5, 1, 2, 3, 4, and 5%. After treatment, cell viability was determined using the CCK-8 assay by measuring the absorbance at 450 nm. This was performed to evaluate the effects of CCPJ and DSS on Caco-2 cell viability and to determine the appropriate concentrations for modeling and drug administration.

### 2.9 Establishment of the cellular inflammation model

Based on the results of the CCK-8 assay, the treatment concentrations were determined. The experimental groups were set up as follows: the Blank Control (BC) group received complete culture medium; the Model Group (MG) was treated with 4% DSS; the Low-concentration CCPJ group (100) received 100 μg/mL CCPJ + 4% DSS; the Medium-concentration CCPJ group (200) received 200 μg/mL CCPJ + 4% DSS; and the High-concentration CCPJ group (400) received 400 μg/mL CCPJ + 4% DSS. Except for the blank control group, all groups were first treated with 4% DSS for 24 h, after which the medium was replaced with corresponding medium containing the indicated concentration of CCPJ for another 24 h before subsequent assays were performed.

### 2.10 Determination of intracellular LDH and SOD content in Caco-2 cells

Caco-2 cells were seeded in 96-well plates at a density of 2 × 10^5^ cells/mL (100 μL per well) and cultured for 24 h. The cellular inflammation model was subsequently established according to the method described in Section 2.9. Following modeling, the levels of LDH and SOD in different treatment groups were measured strictly in accordance with the manufacturer’s instructions provided in the respective LDH and SOD assay kits.

### 2.11 Secretion of cytokines by Caco-2 cells

Cells in the logarithmic growth phase were seeded in 6-well plates at a density of 10^6^ cells/mL (2 mL per well) and cultured for 48 h. The cellular inflammation model was then established according to the protocol described in Section 2.9. Following the modeling process, the concentrations of TNF-*α* and IL-6 in the cell culture supernatant were measured to assess cytokine release, strictly following the manufacturer’s instructions provided with the respective TNF-α and IL-6 ELISA kits.

### 2.12 Detection of ROS and mitochondrial ROS

Cells in the logarithmic growth phase were seeded in 6-well plates at a density of 10^6^ cells/mL (2 mL per well) and cultured for 48 h. The cellular inflammation model was established according to the method described in Section 2.9. Subsequently, the cells were digested with trypsin and collected into 1.5 mL brown microcentrifuge tubes. The harvested cells were incubated with specific fluorescent probes for ROS and mtROS at 37 °C for 30 min. After incubation, the cells were washed three times with phosphate-buffered saline (PBS). The fluorescence intensity was immediately analyzed using flow cytometry. Concurrently, the cells were observed under an inverted fluorescence microscope, and images were captured using its integrated photography system.

### 2.13 Detection of mitochondrial membrane potential

Caco-2 Cells in the logarithmic growth phase were seeded in 6-well plates at a density of 10^6^ cells/mL (2 mL per well) and cultured for 48 h. The cellular inflammation model was established according to the method described in Section 2.9.

The JC-1 staining working solution was prepared as follows: for each well of a 6-well plate, 1 mL of working solution was required. JC-1 (200X) was diluted with ultrapure water at a ratio of 1:160. After thorough dissolution and mixing, 2 mL of JC-1 staining buffer (5X) was added and mixed well to obtain the final working solution. After aspirating the culture medium from the 6-well plates, the cells were gently rinsed once with PBS. Then, 1 mL of antibiotic-free cell culture medium was added to each well, followed by the addition of 1 mL of the freshly prepared JC-1 working solution. The plates were gently swirled to ensure mixing and incubated at 37 °C for 20 min in a cell culture incubator. During the incubation period, the JC-1 staining buffer (1X) was prepared by diluting the JC-1 staining buffer (5X) with distilled water at a ratio of 1:4, and kept on ice. After the 37 °C incubation, the supernatant was carefully removed. The cells were washed twice with the ice-cold JC-1 staining buffer (1X), using 1 mL per wash. Subsequently, 2 mL of antibiotic-free cell culture medium was added to each well. The fluorescence shift from red (aggregates) to green (monomers) was observed under an inverted fluorescence microscope. Fluorescence intensity was quantified and analyzed using ImageJ software.

### 2.14 Statistical analysis

Statistical analysis and graphing were performed using GraphPad Prism and Origin software. All measured data are expressed as the mean ± standard deviation (^−^x ± s). Normality tests and homogeneity of variance tests were conducted on the dataset. If the data followed a normal distribution and exhibited homogeneity of variance, a one-way analysis of variance (ANOVA) was applied, followed by the Least Significant Difference (LSD) *post hoc* test for multiple comparisons between groups. If the data violated the assumption of normality, non-parametric tests were used instead. *p*-value of less than 0.05 (*p* < 0.05) was considered statistically significant.

## 3 Results

### 3.1 Extraction yield and total sugar content of crude CCPJ prior to purification

The crude polysaccharide extraction yield of CCPJ-40 was 1.2 ± 0.05%, with a total sugar content of 351 ± 5 mg/g; CCPJ-60 yielded 1.1 ± 0.05% with a total sugar content of 243 ± 5 mg/g; while CCPJ-80 showed the highest extraction yield at 1.5 ± 0.05% and total sugar content of 360 ± 5 mg/g.

### 3.2 Monosaccharide composition of CCPJ

**Figure 1:**
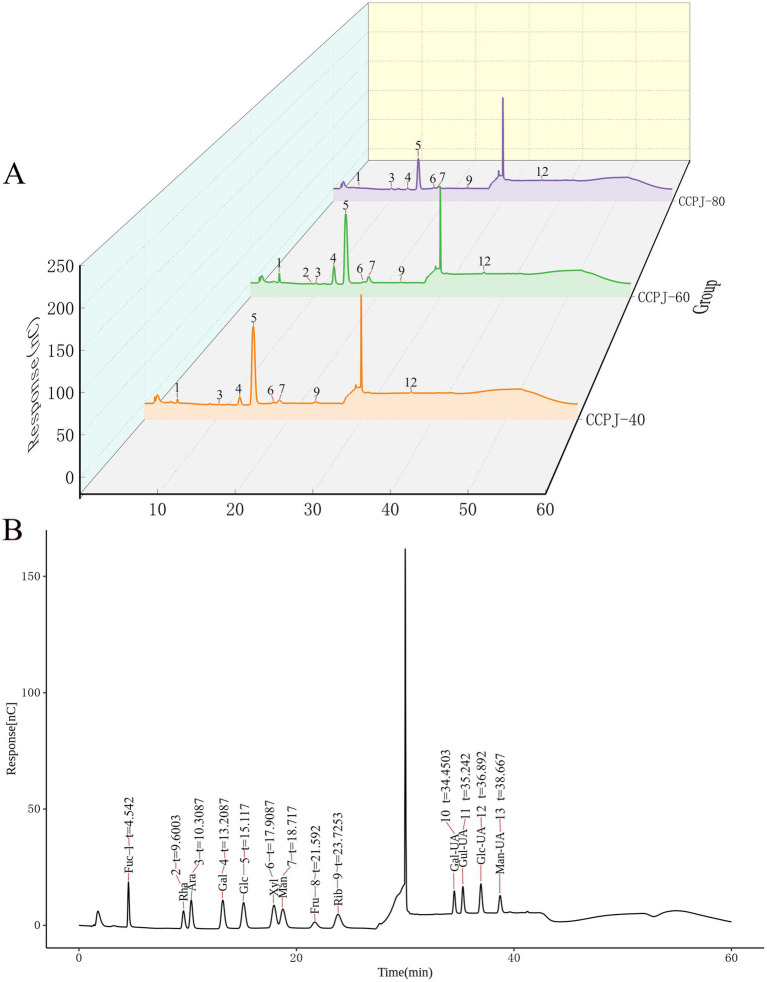
Monosaccharide composition **(A)** Chromatogram of the sample; **(B)** Chromatogram of monosaccharide standards.

As shown in Figure 1, the main components of both CCPJ-40 and CCPJ-80 were L-fucose, L-arabinose, galactose, glucose, xylose, mannose, ribose, and D-glucuronic acid. In contrast, CCPJ-60 contained rhamnose in addition to all the monosaccharides present in CCPJ-40 and CCPJ-80, including L-fucose, L-arabinose, galactose, glucose, xylose, mannose, ribose, and D-glucuronic acid (For details, see Appendix 1).

**Table A1:** Form 1 monosaccharide composition.

样品 名称	Fuc ug/mg	Ara ug/mg	Rha ug/mg	Gal ug/mg	Glc ug/mg	Xyl ug/mg	Man ug/mg	Rib ug/mg	Glc-UA ug/mg
CCPJ- 40	11.592	2.4489	0	33.0696	451.5075	6.1	25.472	13.0655	7.6692
CCPJ- 60	33.1313	5.7474	3.8969	92.4626	501.6874	6.5415	60.0159	5.0765	14.0806
CCPJ- 80	1.787	8.5173	0	8.2684	204.7734	7.606	17.0124	3.4707	2.8108

### 3.3 Absolute molecular weight and molecular chain conformation of CCPJ

**Figure 2:**
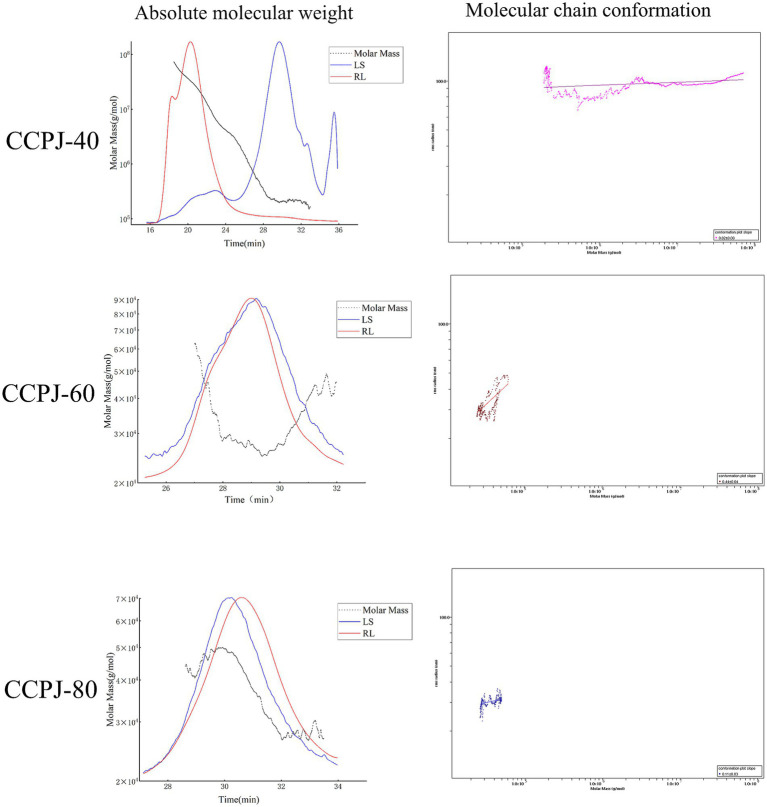
Absolute molecular weight and molecular chain conformation of CCPJ.

As shown in Figure 2, the polydispersity indices (Mw/Mn) of CCPJ-40, CCPJ-60, and CCPJ-80 were 11.240, 1.034, and 1.051, respectively; their absolute molecular weights were 3308.443 kDa, 30.706 kDa, and 37.092 kDa, respectively. In the molecular chain conformation analysis, the slope values for CCPJ-40, CCPJ-60, and CCPJ-80 were 0.02 ± 0.00, 0.44 ± 0.04, and 0.11 ± 0.03, respectively (For details, see Appendix 2).

**Table A2:** Form 2 absolute molecular weight of polysaccharides.

样品名称	Polydispersity (Mw/Mn)	Mw (kDa)	Mn (kDa)
CCPJ-40	11.240	3308.443	294.337
CCPJ-60	1.034	30.706	29.703
CCPJ-80	1.051	37.092	35.279

### 3.4 Fourier transform infrared spectroscopy

**Figure 3:**
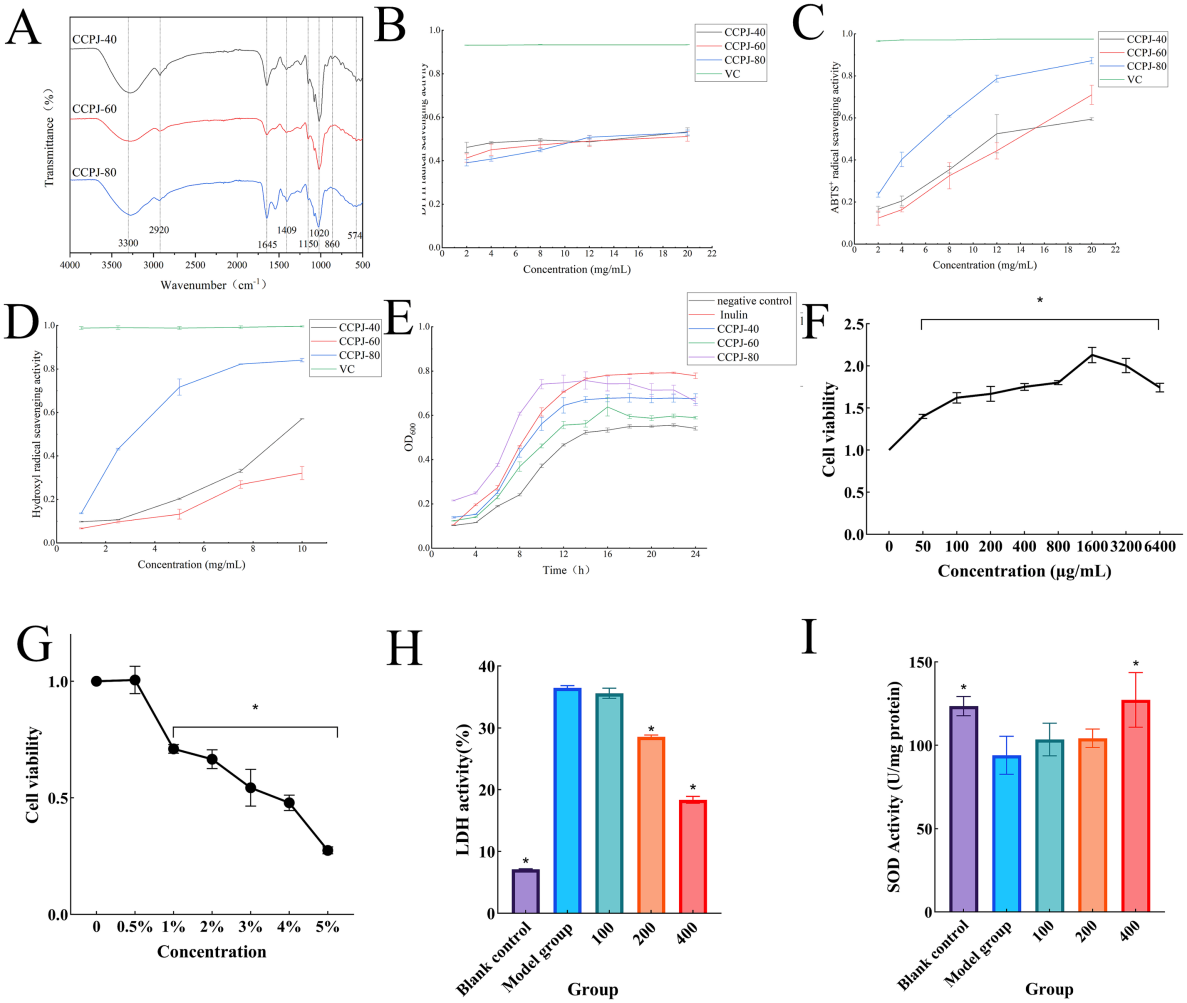
**(A)** FTIR spectra of CCPJ; **(B)** DPPH radical scavenging rate; **(C)** ABTS^+^• radical scavenging rate; **(D)** Hydroxyl radical scavenging rate; **(E)** Growth curves of *Lactobacillus plantarum* supplemented with different CCPJ fractions; **(F)** Effects of different concentrations of CCPJ-80 on Caco-2 cell viability; **(G)** Effects of different concentrations of DSS on Caco-2 cell viability; **(H)** Effects of CCPJ on LDH levels in DSS-induced inflamed Caco-2 cells; **(I)** Effects of CCPJ on SOD levels in DSS-induced inflamed Caco-2 cells. Data are presented as mean ± SD (n = 3). *p < 0.05 vs. the model group.

As shown in Figure 3A, the FTIR spectra of CCPJs obtained by ethanol precipitation at different concentrations are presented. Characteristic absorption peaks were observed at the following wavenumbers: 3300–3,250 cm^−1^, 2,950–2,900 cm^−1^, 1,645–1,640 cm^−1^, 1,400–1,410 cm^−1^, 1,200–1,000 cm^−1^, 870–860 cm^−1^, and 570–560 cm^−1^.

### 3.5 Antioxidant activity

As shown in Figure 3B, at polysaccharide concentrations ranging from 2 to 20 mg/mL, the DPPH radical scavenging rates of CCPJ-40, CCPJ-60, and CCPJ-80 were 53, 51, and 53%, respectively. Figure 3C demonstrates that within the same concentration range (2–20 mg/mL), the ABTS^+^• radical scavenging rates of CCPJ-40, CCPJ-60, and CCPJ-80 increased with rising polysaccharide concentration, showing a significant positive correlation, reaching 59, 70, and 87%, respectively. As illustrated in Figure 3D, at concentrations of 1–10 mg/mL, the hydroxyl radical scavenging rates of CCPJ-40, CCPJ-60, and CCPJ-80 were 57, 32, and 84%, respectively.

### 3.6 Growth promotion of *Lactobacillus plantarum*

As shown in the Figure 3E, the growth of *Lactobacillus plantarum* exhibited an increasing trend followed by stabilization when supplemented with CCPJ-40 or CCPJ-60. While the growth pattern of cultures supplemented with CCPJ-80 was similar to that of CCPJ-40 and CCPJ-60 groups during the first 0–12 h, and even demonstrated a faster growth rate, it showed a declining trend after 12 h.

### 3.7 Effects of different concentrations of CCPJ and DSS on cell viability

In this experiment, a Caco-2 cell validation model was established using DSS. After treating the cells with different concentrations of DSS (0, 0.5, 1, 2, 3, 4, 5%), as shown in Figure 3G, the comparison of cell viability revealed that concentrations of 1 to 5% all had a significant inhibitory effect on cell survival rate (*p* < 0.05). Therefore, based on the literature (7), a DSS concentration of 4% was selected to establish the cellular inflammation model. In preliminary experiments, CCPJ-80 demonstrated superior physicochemical and activity indicators; consequently, CCPJ-80 was chosen for subsequent experiments. After treating the cells with different concentrations of CCPJ-80 (0, 50, 100, 200, 400, 800, 1,600, 3,200, 6,400 μg/mL), the cell viability results are shown in Figure 3F. Concentrations of 50–1,600 μg/mL CCPJ-80 significantly promoted cell viability (*p* < 0.05). However, when the concentration of CCPJ-80 exceeded 1,600 μg/mL, the cell viability gradually decreased. Thus, concentrations of 100, 200, and 400 μg/mL CCPJ-80 were selected as the intervention groups for subsequent experiments.

### 3.8 Determination of LDH and SOD content in Caco-2 cells

Lactate dehydrogenase (LDH) is a stable soluble cytosolic enzyme present in cells. When cell membrane damage occurs, LDH is rapidly released from the cytoplasm, making it a marker in cytotoxicity studies (31). As shown in Figure 3H, LDH levels were significantly increased (*p* < 0.05) in DSS-treated cells compared to the control. Treatment with 100 μg/mL CCPJ-80 showed no significant difference in LDH levels compared to the model group. However, when the concentration of CCPJ-80 was increased to 200 and 400 μg/mL, LDH levels decreased significantly (*p* < 0.05), with higher CCPJ-80 concentrations resulting in lower LDH content, suggesting a dose-dependent protective effect.

Superoxide dismutase (SOD) is a key antioxidant enzyme that maintains cellular redox homeostasis by scavenging excess oxygen free radicals (7). As shown in Figure 3I, SOD content was significantly decreased (*p* < 0.05) in the model group compared to the blank control group. Treatment with 100 and 200 μg/mL CCPJ-80 showed no significant difference in SOD content compared to the model group, although an increasing trend was observed. When the CCPJ-80 concentration was increased to 400 μg/mL, SOD content was significantly higher (*p* < 0.05) compared to the model group, indicating that a higher concentration of CCPJ-80 effectively restored SOD activity.

### 3.9 Determination of cytokine (TNF-*α* and IL-6) levels in Caco-2 cells

**Figure 4:**
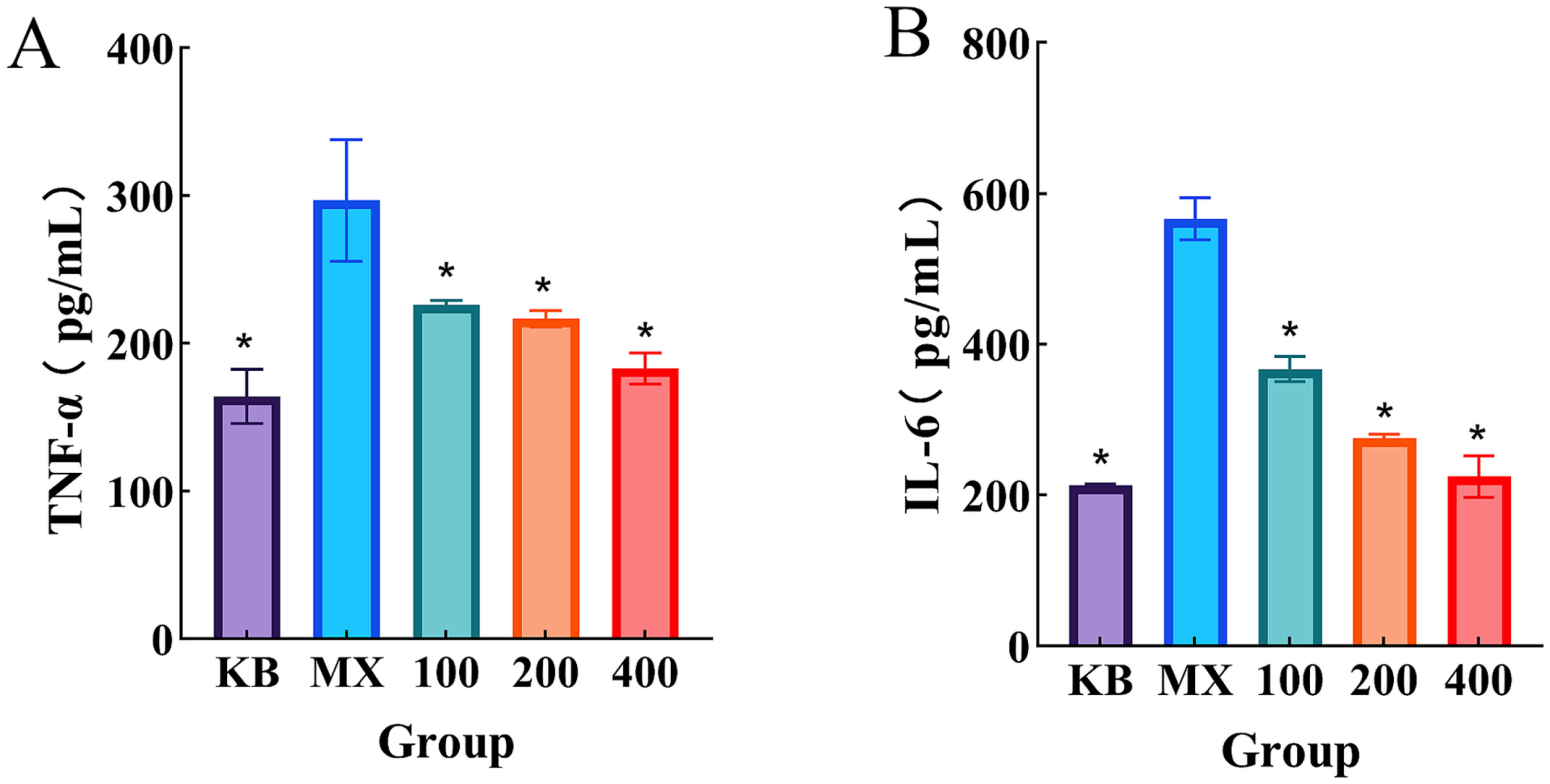
**(A)** Levels of TNF-α in Caco-2 cells; **(B)** Levels of IL-6 in Caco-2 cells. Data are presented as mean ± SD (*n* = 3). *p < 0.05 versus the model group.

Studies have demonstrated that polysaccharide treatment can suppress the expression of inflammatory cytokines such as TNF-α and IL-6. As shown in Figure 4, compared with the blank control group, the levels of TNF-α and IL-6 were significantly increased (*p* < 0.05) in the model group. In contrast, treatment with polysaccharides significantly reduced the levels of TNF-α and IL-6 in Caco-2 cells compared to the model group (*p* < 0.05).

### 3.10 Detection of ROS levels in Caco-2 cells

**Figure 5:**
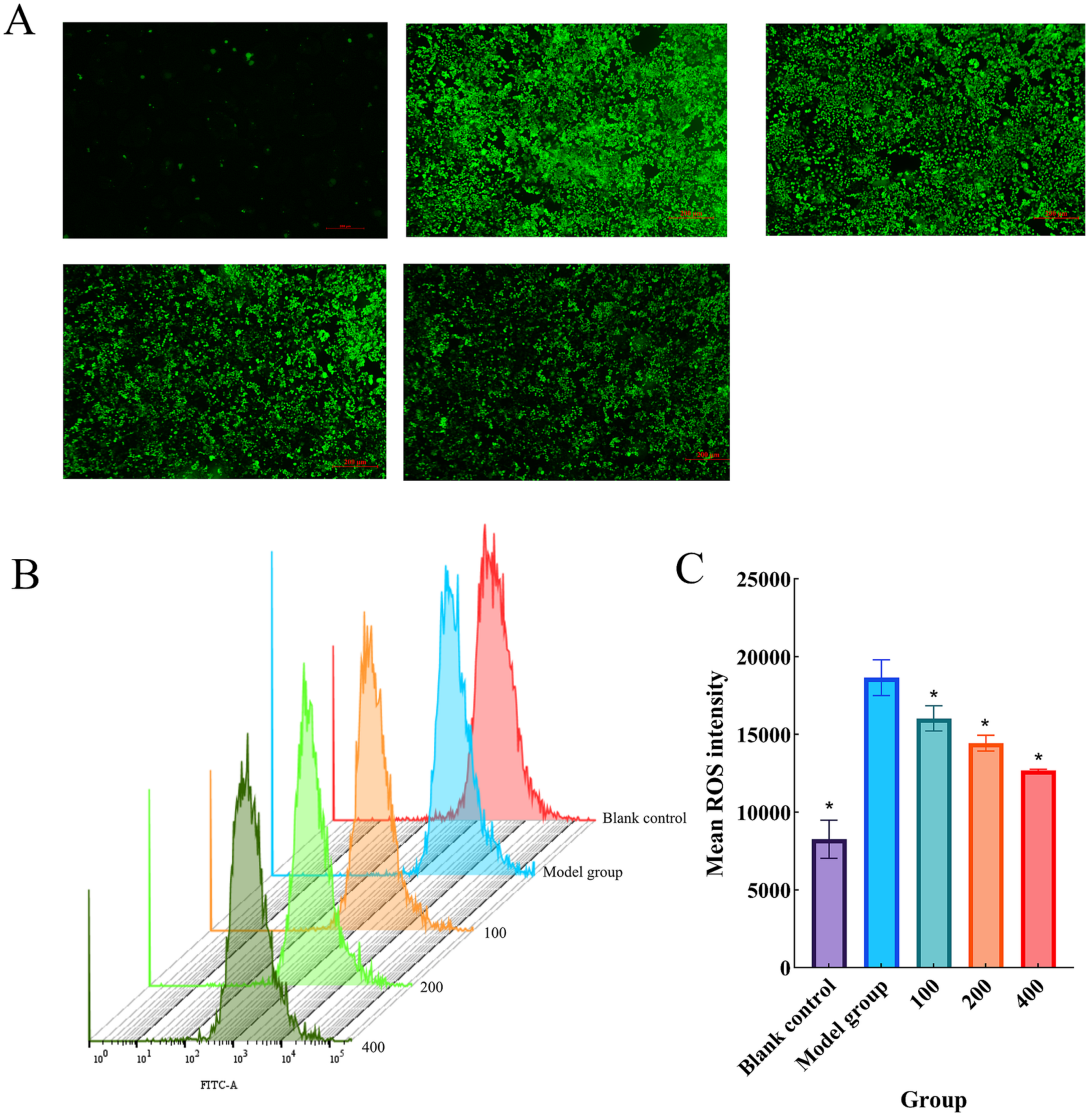
Detection of ROS levels in Caco-2 cells. **(A)** Representative images of ROS detection in Caco-2 cells by inverted fluorescence microscopy; **(B)** Flow cytometric analysis of ROS levels in Caco-2 cells; **(C)** Quantitative analysis of ROS levels. Data are presented as mean ± SD (*n* = 3). *p < 0.05 versus the model group.

ROS play important roles in cellular signaling and tissue homeostasis, where maintaining ROS balance is crucial as disruption of this balance can lead to disease development (32). Fluorescence detection of intracellular ROS was performed and representative images are shown in Figure 5A. Furthermore, flow cytometric analysis was conducted for validation Figure 5B. Quantitative analysis of ROS based on FITC-A fluorescence intensity Figure 5C revealed that ROS levels were significantly increased in the model group compared to the blank control group (*p* < 0.05). Treatment with CCPJ-80 resulted in a significant reduction in fluorescence intensity (*p* < 0.05) in a concentration-dependent manner, with higher concentrations leading to greater decreases in ROS levels.

### 3.11 Detection of mt ROS levels in Caco-2 cells

**Figure 6:**
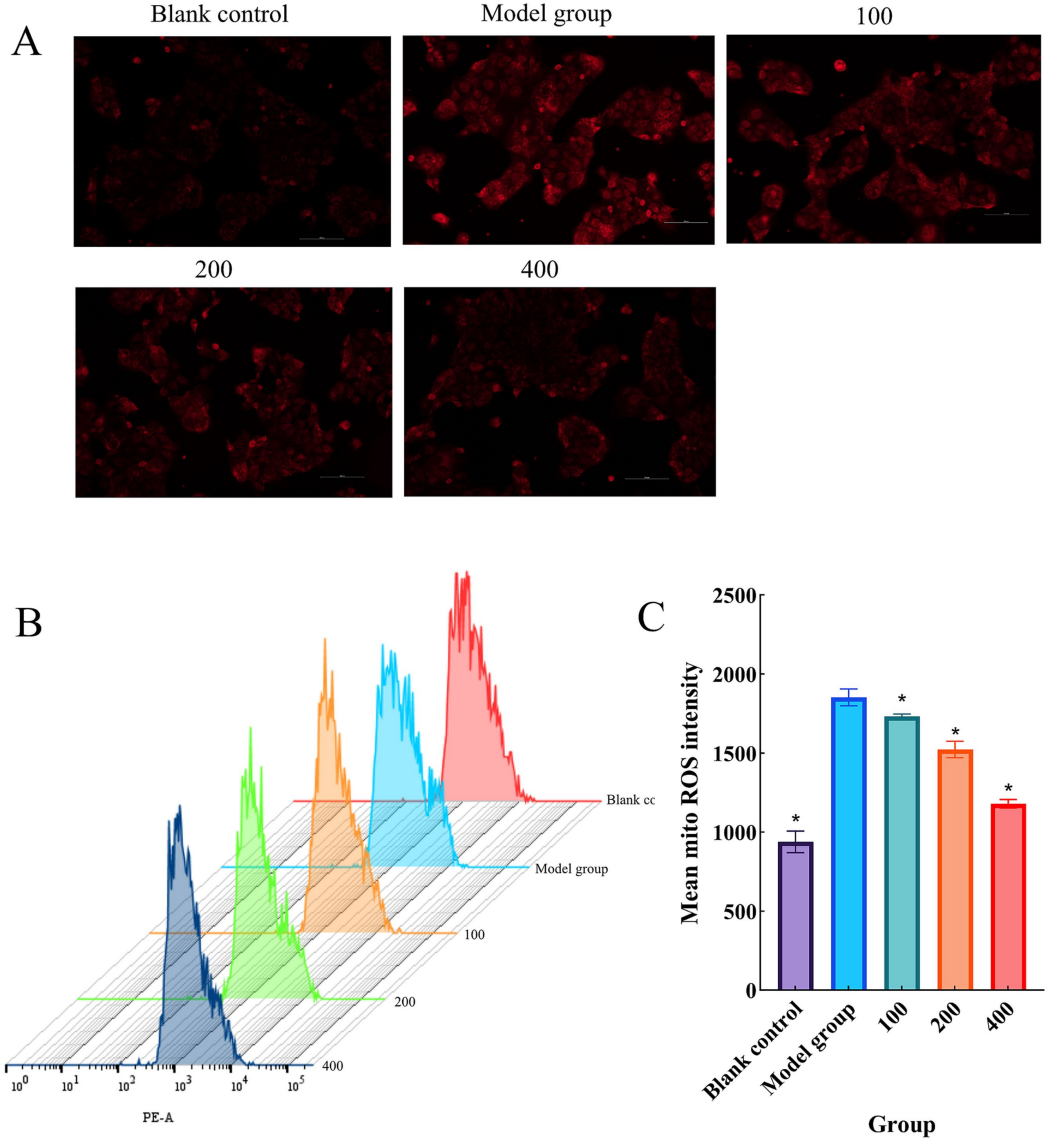
mt ROS levels in Caco-2 cells. **(A)** Representative images of mt ROS detection by inverted fluorescence microscopy; **(B)** Flow cytometric analysis of mt ROS levels; **(C)** Quantitative analysis of mt ROS levels. Data are presented as mean ± SD (*n* = 3). *p < 0.05 versus the model group.

Excessive ROS production primarily originates at the mitochondrial level, leading to oxidative stress and impaired mitochondrial function, which consequently elevates the level of mt ROS (33). This study preliminarily observed the effects of CCPJ on mitochondria by detecting mt ROS levels using inverted fluorescence microscopy and flow cytometry. As shown in Figure 6, the results showed that compared with the blank control group, mt ROS levels were significantly increased in the model group (*p* < 0.05). After treatment with CCPJ, mt ROS levels were significantly reduced (*p* < 0.05) in a concentration-dependent manner.

### 3.12 Detection of mitochondrial membrane potential in Caco-2 cells

**Figure 7:**
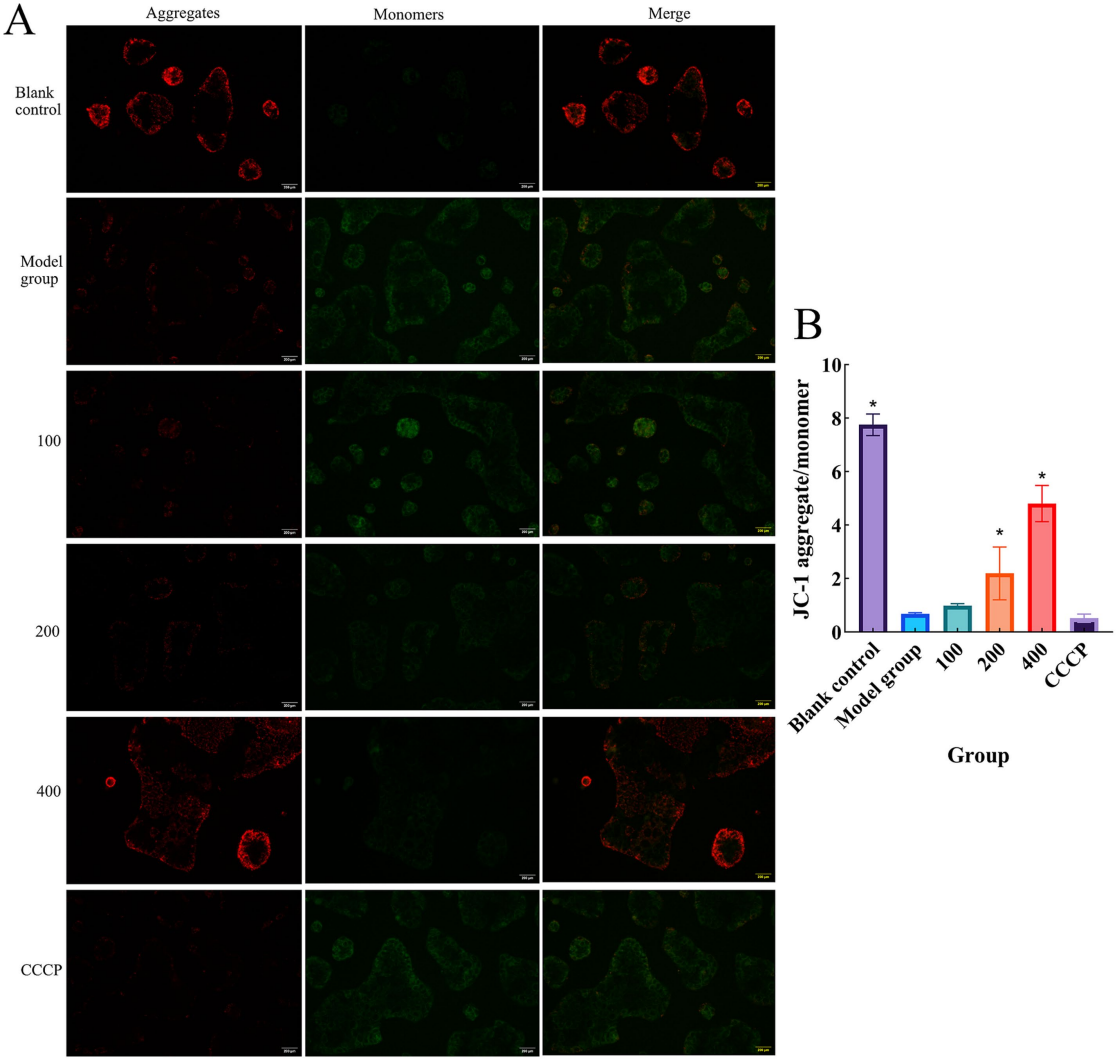
Detection of Mitochondrial Membrane Potential **(A)** Representative images of JC-1 staining in Caco-2 cells (scale bar = 100 μm); **(B)** Quantitative analysis of JC-1 fluorescence. Data are presented as mean ± SD (*n* = 3). *p < 0.05 versus the model group.

In this study, the mitochondrial membrane potential (MMP) was detected using the JC-1 fluorescent probe, and changes in fluorescence were observed under an inverted fluorescence microscope. High MMP generates red fluorescence, while low MMP produces green fluorescence. As shown in Figure 7, the change in MMP was assessed by measuring the red-to-green fluorescence ratio to evaluate the effect of CCPJ-80 on mitochondrial function. The results revealed that after treatment with 4% DSS, Caco-2 cells showed enhanced green fluorescence and weakened red fluorescence. Compared with the blank control group, the red-to-green fluorescence ratio decreased significantly. However, in the groups treated with different concentrations of CCPJ-80, the fluorescence signals and their ratio in the 100 μg/mL CCPJ-80 group showed no significant difference compared with the model group. In the 200 μg/mL CCPJ-80 group, the red fluorescence signal showed no obvious change, but the green fluorescence was weakened, resulting in a significant difference in the red-to-green fluorescence ratio compared with the model group (*p* < 0.05). In the 400 μg/mL CCPJ-80 group, the red fluorescence signal was enhanced, and the green fluorescence was weakened, showing a highly significant difference in the red-to-green fluorescence ratio compared with the model group (*p* < 0.05).

## 4 Discussion

The present study successfully separated crude CCPJ into three fractions (CCPJ-40, −60, and −80) using graded ethanol precipitation, a method that exploits the differential solubility of polysaccharides in varying ethanol concentrations. Although the extraction yields of the fractions were similar, their structural characteristics and bioactivities exhibited significant differences. These findings are consistent with those reported by Chen (34) et al., confirming that fractional ethanol precipitation is an effective method for separating heterogeneous polysaccharide mixtures.

Monosaccharide composition analysis provided key evidence for the effectiveness of the fractionation. CCPJ-40 and CCPJ-80 shared similar major monosaccharide profiles, while CCPJ-60 uniquely contained rhamnose. Research indicates that the presence of rhamnose is often associated with a specific class of pectic polysaccharides rich in rhamnose and galacturonic acid, which typically exhibit distinct molecular conformations, branching patterns, and polarity characteristics (35). This may explain why CCPJ-60 reached its solubility threshold and precipitated specifically at the 60% ethanol concentration. These results further indicate that fractional ethanol precipitation can effectively separate crude polysaccharides based on differences in their chemical structures, which macroscopically reflect variations in chemical composition.

In-depth analysis of polysaccharide molecular conformation and homogeneity revealed key physicochemical characteristics of each fraction. Multi-angle laser light scattering allows for the investigation of molecular configuration by analyzing double logarithmic plots. Studies have shown that in a plot with log (Molar Mass) as the abscissa and log(R. M. S. Radius) as the ordinate, the slope of the resulting line reveals the conformational characteristics of the polymer: a slope of 1.8–2.0 exhibit a rod-like conformation, those with a slope of 0.5–0.8 adopt a random coil conformation, and molecules with a slope of 0–0.3 assume a highly branched, tightly coiled spherical conformation (36). The present study revealed that CCPJ-60 exhibits a lightly branched, chain-like structure, while CCPJ-80 displays a branched and tightly coiled, globular architecture. Notably, the exceptionally low slope value observed for CCPJ-40 likely reflects its distinct physical properties, indicating a highly branched, tightly coiled spherical conformation. These findings further demonstrate that fractional ethanol precipitation can effectively separate polysaccharides based on differences in their molecular structure, such as the degree of branching. Polydispersity analysis further indicated that the CCPJ-40 fraction, precipitated with 40% ethanol, was highly heterogeneous, while the fractions precipitated with 60 and 80% ethanol showed good homogeneity. The absolute molecular weight results align with the general rule that lower ethanol concentrations precipitate high molecular weight polysaccharides, whereas higher concentrations precipitate lower molecular weight ones (29). Some studies suggest that lower molecular weight polysaccharides may possess a higher density of active groups (37). These structural features may collectively contribute to the stronger bioactivity subsequently demonstrated by CCPJ-80.

Fourier transform infrared spectroscopy further revealed the fine structural similarities and differences among the fractions. All fractions exhibited characteristic absorption peaks typical of polysaccharides. The broad absorption band at 3300–3250 cm^−1^ was attributed to O-H stretching vibrations (38), while the peak at 2950–2900 cm^−1^ originated from C-H stretching vibrations (39); these two characteristic peaks collectively confirmed the saccharide nature of CCPJ. The absorption at approximately 1,645 cm^−1^ corresponds to free carboxyl groups (1700–1,600 cm^−1^), and the peak at 1409 cm^−1^ can be ascribed to symmetric COO^−^ stretching (1400–1,300 cm^−1^) (40). The complex absorption peaks between 1,200–1,000 cm^−1^ represent the characteristic region for vibrations of C-O-C and C-O-H bonds in sugar rings (41), and these signals indicate the presence of pyranose ring structures in the polysaccharide fractions (42). Notably, the absorption peak intensities at both 1,645 cm^−1^ and 1,409 cm^−1^ for CCPJ-80 were significantly stronger than those of the other fractions, clearly indicating a higher proportion of uronic acids (42). These key structural differences, particularly the higher uronic acid content in CCPJ-80, are considered to be an important potential factor contributing to the variations in its biological activity.

The results from *in vitro* antioxidant assays, including ABTS^+^•, DPPH, and hydroxyl radical scavenging activities, were highly consistent, demonstrating that CCPJ-80 exhibited significantly superior activity compared to CCPJ-40 and CCPJ-60. This enhanced activity may be attributed to its higher uronic acid content, as the carboxyl and COO^−^ groups can act as efficient hydrogen or electron donors, effectively neutralizing various free radicals (43). In the *Lactobacillus plantarum* growth promotion assay, CCPJ-80 again demonstrated the most significant growth-enhancing effect, consistent with its properties as a high-quality prebiotic—i.e., resistant to host digestion but readily utilized by specific beneficial bacteria (44). However, after 12 h of rapid growth, the growth curve of *L. plantarum* in the CCPJ-80 group began to decline. This may be attributed to the preferential and rapid consumption of CCPJ-80, leading to a carbon-depleted, oligotrophic state in the medium during the later phase (45). Concurrently, the high metabolic activity during the exponential growth phase resulted in substantial accumulation of bacterial metabolites, which often exert inhibitory effects on growth (46). Therefore, the CCPJ-80 group, which exhibited the fastest growth and highest biomass accumulation earlier, experienced more pronounced nutrient limitation and metabolic inhibition once the carbon source was exhausted, ultimately leading to the observed decline in growth. Based on its outstanding performance in both antioxidant and prebiotic activities, CCPJ-80 was selected for subsequent cell experiments.

To investigate the protective effect of CCPJ-80 on intestinal barrier function, this study established a dextran sulfate sodium (DSS)-induced inflammatory model using Caco-2 cells. Caco-2 cells spontaneously differentiate into a model exhibiting characteristics of intestinal epithelial cells during *in vitro* culture and are widely used for studying intestinal barrier function and related inflammation (47). Cytotoxicity assays indicated that CCPJ-80 itself was non-toxic to the cells and, when administered alone, even enhanced cell viability. After establishing the Caco-2 cell inflammatory model with 4% DSS and subsequent polysaccharide intervention, the release of lactate dehydrogenase (LDH) was measured to assess cell membrane integrity, as LDH is released into the supernatant upon membrane damage, serving as a key indicator of cellular injury (48). This study found that medium and high concentrations of CCPJ-80 significantly counteracted the cytotoxicity induced by DSS.

The inflammatory response in DSS-induced Caco-2 cells is largely mediated by pro-inflammatory cytokines such as TNF-*α* and IL-6 (49). The results of this study demonstrated that DSS stimulation significantly upregulated the secretion of these two key inflammatory cytokines, whereas CCPJ-80 treatment inhibited their production in a dose-dependent manner. Subsequent investigation into the underlying mechanism revealed that this potent anti-inflammatory effect was closely associated with its antioxidant capacity. DSS stimulation disrupts cellular homeostasis, leading to a sharp increase in intracellular ROS levels (50). Excessive accumulation of ROS can further activate inflammatory signaling pathways, resulting in the substantial secretion of cytokines (51) and thereby inducing oxidative stress (52). This study confirmed that CCPJ-80 significantly reduced the overall intracellular ROS levels in inflamed cells and enhanced the activity of endogenous antioxidant enzymes such as SOD.

Given that mitochondria are central organelles for intracellular ROS production and stress perception, and their dysfunction is a key trigger for cell death (53), this study further investigated the protective effects of CCPJ-80 on mitochondrial function. The results demonstrated that CCPJ-80 treatment effectively suppressed mt ROS generation. Moreover, medium and high concentrations of CCPJ-80 significantly stabilized the mitochondrial membrane potential, which had been reduced by DSS stimulation. Given its relatively high molecular weight (approximately 37 kDa), CCPJ-80 is more likely to exert the aforementioned protective effects by binding to pattern recognition receptors on the cell surface—such as Toll-like receptor 4 (TLR4), which is highly expressed on intestinal epithelial cells—thereby indirectly modulating intracellular signaling pathways. Existing studies indicate that fungal polysaccharides can initiate downstream signal transduction through such receptors: pattern recognition receptors, particularly TLR4, are common targets mediating the bioactivity of polysaccharides (54). For instance, it has been reported that fungal polysaccharides can exert immunomodulatory effects via the TLR4/MyD88 signaling pathway, ultimately suppressing the expression of pro-inflammatory factors such as TNF-α (54), which aligns with the findings of the present study. On the other hand, activation of survival signaling pathways downstream of the same receptor, such as PI3K/Akt, has been shown to directly stabilize mitochondrial membrane potential and reduce mitochondrial ROS production (55). Therefore, we speculate that CCPJ-80 may, through a similar receptor-mediated mechanism, concurrently inhibit inflammatory responses and activate mitochondrial protective signals, thereby synergistically alleviating the interaction cycle between oxidative stress and inflammation. This proposed mechanistic link points the way for future in-depth investigations into its precise receptor targets and signaling networks.

It should be noted that while this study revealed differences in the macroscopic physicochemical properties among the fractions and correlated them with bioactivity, the absence of methylation analysis and nuclear magnetic resonance spectroscopic data precludes establishing a definitive causal relationship between specific structural features—such as glycosidic linkage types and backbone architecture—and the observed biological activities. Given its relatively high uronic acid content, distinct solution conformation, and pronounced bioactivity, CCPJ-80 is likely to be classified as a bioactive polysaccharide. However, its precise structural classification—for example, whether it represents a typical *β*-glucan—requires further confirmation through the detailed structural analyses mentioned above. Future studies will employ methylation analysis (GC–MS) and one/two-dimensional nuclear magnetic resonance spectroscopy (e.g., ^1^H, ^13^C, HSQC) to elucidate the glycosidic linkage types (e.g., distinguishing β-(1 → 3)-glucans from *α*-(1 → 4)-glycogen), branching patterns, and repeating unit structures of fractions such as CCPJ-80. This will enable the establishment of a more precise structure–activity relationship model and utilize the functional model established in this study to further validate its related mechanisms for improving intestinal barrier function at the animal level.

## Data Availability

The raw data supporting the conclusions of this article will be made available by the authors, without undue reservation.
